# Post-Aire Medullary Thymic Epithelial Cells and Hassall’s Corpuscles as Inducers of Tonic Pro-Inflammatory Microenvironment

**DOI:** 10.3389/fimmu.2021.635569

**Published:** 2021-04-02

**Authors:** Martti Laan, Ahto Salumets, Annabel Klein, Kerli Reintamm, Rudolf Bichele, Hedi Peterson, Pärt Peterson

**Affiliations:** ^1^ Molecular Pathology Research Group, Institute of Biomedicine and Translational Medicine, University of Tartu, Tartu, Estonia; ^2^ Institute of Computer Science, Faculty of Science and Technology, University of Tartu, Tartu, Estonia

**Keywords:** Hassall’s corpuscles, medullary thymic epithelial cells, AIRE, thymus, central tolerance, S100A8, S100A9, TLR4 – Toll-like receptor 4

## Abstract

While there is convincing evidence on the role of Aire-positive medullary thymic epithelial cells (mTEC) in the induction of central tolerance, the nature and function of post-Aire mTECs and Hassall’s corpuscles have remained enigmatic. Here we summarize the existing data on these late stages of mTEC differentiation with special focus on their potential to contribute to central tolerance induction by triggering the unique pro-inflammatory microenvironment in the thymus. In order to complement the existing evidence that has been obtained from mouse models, we performed proteomic analysis on microdissected samples from human thymic medullary areas at different differentiation stages. The analysis confirms that at the post-Aire stages, the mTECs lose their nuclei but maintain machinery required for translation and exocytosis and also upregulate proteins specific to keratinocyte differentiation and cornification. In addition, at the late stages of differentiation, the human mTECs display a distinct pro-inflammatory signature, including upregulation of the potent endogenous TLR4 agonist S100A8/S100A9. Collectively, the study suggests a novel mechanism by which the post-Aire mTECs and Hassall’s corpuscles contribute to the thymic microenvironment with potential cues on the induction of central tolerance.

## Introduction

The thymus is a primary immune organ required for T cell development. The maturation process of the developing T cells, the thymocytes, involves somatic recombinations to randomly generate functional T cell receptors that in principle can recognize all possible antigenic determinants [reviewed in (1)]. Thus, in order to avoid the escape of potentially harmful, self-reactive T cell clones, these clones need to be either eliminated physically or changed functionally before their exit from the thymus. These processes, collectively known as central tolerance induction, take place in the thymic medulla and comprise negative selection and thymic regulatory T cell (Treg) induction. Both negative selection and Treg differentiation are believed to rely on T-cell receptor (TCR)-derived signal strength (2), which in turn depends on the availability and affinity of self-peptides in the thymus and is regulated by a range of co-stimulatory molecules expressed on antigen presenting cells (APC) (1, 2). Therefore, the development of efficient and self-tolerant T-cells depends on complex interactions between a range of different thymic cell types and is shaped by the local microenvironment (3).

As opposed to all other organs, a specific feature of the thymic microenvironment is a constitutive low-grade expression of proinflammatory mediators, inflammatory cytokines and chemokines even under physiological conditions (4–6) (covered in detail below). There is accumulating evidence that this low-grade inflammatory signaling may play a role in thymocyte development. Indeed, the recent data suggest that the tonic inflammation can affect the final stages of single positive T cell development as well as thymic Treg generation *via* mobilization of thymic dendritic cells (7, 8). Hence, the tonic pro-inflammatory microenvironment in the thymus has the potential to affect central tolerance induction and to shape the resulting repertoire of peripheral T cells and Tregs. The cellular and molecular mechanisms leading to this unique phenomenon, however, are not fully understood. Below we will summarize the current knowledge together with some novel evidence that these inflammatory signals are provided by the medullary thymic epithelial cells (mTECs) at the very late stages of their differentiation.

## Central Tolerance Induction and mTEC Differentiation

Although the developing thymocytes comprise the majority of the cellular mass of the thymus, their proper development is directed by the non-hematopoietic thymic stroma including the thymic epithelial cells and fibroblasts as well as by the non-thymocyte hematopoietic compartment including the APCs, i.e. the dendritic cells, B cells and macrophages (3). In regard to the induction of central tolerance, a central role is played by the mTECs that have a unique property to express a huge variety of different genes and proteins including the ones whose expression is otherwise restricted to a certain peripheral cell or tissue type (9). This ectopic gene expression is largely controlled by a transcriptional regulator Aire and is critical for the induction of central tolerance to these self-proteins either by directing the self-reactive thymocyte clones to apoptosis (i.e. negative selection) or directing them toward the Treg lineage (10–12). Accordingly, mutations in Aire result in a defect in central tolerance in humans as well as in mice and rats affecting both of these arms of central tolerance (10, 13, 14). In addition to the well-characterized role in ectopic antigen expression, Aire has been proposed to control a number of other functions including regulation of thymic chemokines (15–17) and mTEC maturation (18). Regardless of the precise mechanism, however, the end-result of Aire-deficiency is a defect in central tolerance, which may precipitate in autoimmunity.

Due to the central role of Aire expressing mTECs in thymic tolerance induction, there has been a lot of interest in the Aire+ mTEC lineage differentiation and mTEC differentiation in general [reviewed in (19)]. It is now widely accepted that at least during fetal development both the cortical as well as the medullary epithelial cells are derived from a single bipotent progenitor (20, 21), while the existence of the bipotent progenitor in the adult thymus has remained controversial. After receiving a signal yet to be identified, some of the progenitors are directed toward the mTEC lineage and upregulate the proliferation marker Ki67 (22) to become more populous and can still give rise to different mature mTEC lineages. Recent advances in single-cell transcriptomics in mice (22–26) and humans (27) have highlighted the heterogeneity of these functionally diverse thymic cells and although minor differences exist between the results, most of the respective studies agree that in mice the mature mTECs can be divided into four subpopulations: 1) mTEC I, characterized by the dependency of lymphotoxin (LT)β signaling, high expression of CCL21 and lack of MHCII expression (28, 29); 2) mTEC II, characterized by RANK-dependency and high expression of Aire, MHCII and thousands of tissue-restricted genes (30, 31); 3) mTEC III, known as post-Aire cells or corneocyte-like mTECs, which express low/mid MHCII and whose gene expression profile resembles late-stage keratinocytes/corneocytes (see below); and 4) mTEC IV, known as thymic tuft cells, which express IL-25 and whose gene expression profile resembles intestinal tuft cells (23, 24). Single-cell analysis of the human thymus confirmed these four main mTEC subpopulations but added mTEC-myo and mTEC-neuro as two additional subpopulations present in humans but not in mice (32).

## The Aire+ mTECs, Post-Aire mTECs and Hassall’s Corpuscles

As it is at the Aire+ (mTEC II) stage, where the mTECs express thousands of self-antigens, MHCII and co-stimulatory molecules CD80 and CD86, this population in particular has been profoundly studied as a central player in central tolerance induction. As these cells represent a functionally mature post-mitotic cell population they were, until quite recently, also considered the endpoint of the Aire+ lineage existence. However, several fate-mapping and single cell transcriptomics approaches have identified that the differentiation of mTECs extends beyond the Aire+ differentiation stage to become the mTEC III (25, 31, 33–35). At this post-Aire stage, the cells down-regulate Aire together with most of the Aire-dependent proteins and lose accordingly their ability to express a broad range of ectopic genes. In addition, these cells downregulate the machinery required for direct antigen presentation including MHCII and the co-stimulatory molecules (31, 34) and depend from this point on the APC-mediated cross-presentation to present the expressed proteome to the developing thymocytes. At the same time, the post-Aire cells become enriched for proteins classically associated with end-stage keratinocytes, such as involucrin (Ivl) (36), Lekti (37), and a variety of different keratins (31), obtaining a corneocyte-like phenotype.

In addition to the conventional mTECs described above, the thymic medulla contains unique structures called Hassall’s corpuscles (HCs). These structures, firstly characterized in 1846 by Arthur Hill Hassall (38) in human thymi have, after their initial description, been found in several other mammals (39) but as well as in bird and fish species (40, 41). In humans, the HCs appear as a concentric merged cluster of unnucleated cells with a typical diameter of 20-100 μm and are present in large quantities already by the 28^th^ week of prenatal development (42). As the size and numbers of HCs shrink together with age-related thymic involution (43) and thymic hyperplasia in myasthenia gravis or lymphomas are characterized by increased numbers of HCs (44, 45), their abundance seems to be related with thymic activity. As the size of HCs, on the other hand, correlates with the size of the thymus (39, 46), they are relatively hard to detect in smaller rodents such as mice and usually require antigen-specific immunostainings to visualize (18). Although the function and nature of the HCs have been studied and speculated since their discovery, it has been established only quite recently that these unique structures represent the final differentiation stage of the Aire+ lineage (19). The evidence comes from studies showing retention of Aire reporters in the HCs once the Aire itself has been already down-regulated (34), from studies showing further upregulation of corneocyte-associated proteins in the HCs (18, 34), and are indirectly also reinforced by the fact that the HCs are nearly missing in the Aire KO mouse (18). Further support comes from human studies showing that in thymomas the presence of HCs is restricted to the subtypes with Aire expression (47) and that in patients with Down syndrome, the presence of the third copy of the chromosome 21 (where the AIRE gene is located) results in increased numbers of Aire positive cells together with enlarged Hassall’s corpuscles (48).

Collectively, the current data corroborate the conclusion that following the Aire+ (mTEC II) stage, the differentiation to post-Aire mTECs (mTEC III) and further to the HCs represent the final steps of the Aire+ lineage. Regarding the function of the post-Aire stages, however, the corresponding data is rather scarce. Because of their natural location in the thymic medulla, i.e. the site of central tolerance induction, most of the proposed functions have been related to their specific expression-pattern of self-proteins. For example, post-Aire cells/HCs have been reported to express keratinocyte-restricted and pemphigus related autoantigens Dsg-1 and Dsg-3 (34, 49, 50) but also proinsulin (51), an autoantigen in type 1 diabetes.

## Tonic Inflammatory Signaling, Late-Stage mTECs, and Central Tolerance Induction

Inflammation, characterized by increased production of inflammatory cytokines and chemokines, is a biological response to harmful stimuli such as pathogens and tissue damage. In this sense, the thymus seems to be a unique organ with constitutive production of pro-inflammatory mediators even under physiological conditions (4). A steady-state expression of type 1 interferons (IFN) has been shown in mouse mTECs and dendritic cells using an IFNβ reporter (6) and the secretion of type 1 IFN has been demonstrated in normal human thymi without any pathological stimulus (5). Although the precise upstream stimuli and functional consequences of this phenomenon are still largely unknown, there is increasing evidence that the post-Aire cells and HCs may play a role behind this specific feature of the thymic microenvironment, and that this in turn may modulate the induction of central tolerance. Thus, HCs have been shown to express thymic stromal lymphopoietin (TSLP) (52), which in turn is known for its capability to convert the conventional T cells to Tregs (52, 53) and can accordingly potentially promote the thymic Treg induction. Also, a recent study connected the senescence-like phenotype of HCs to IFNα production from thymic DCs and suggested that the lack of the low-grade inflammatory signaling results in impaired development of single-positive thymocytes (7). Another recent study showed the importance of Toll-like receptors and the resulting MyD88 signaling in mTECs for the expression of several inflammatory cytokines, DC recruitment and Treg induction (8). The evidence supporting the role of post-Aire mTECs and HCs in the induction of the pro-inflammatory microenvironment comes from the Aire KO mouse which, on the one hand, lacks post-Aire cell populations (18) but on the other hand, has reduced expression of several constitutively expressed inflammatory mediators (7) as well as functional defects both in negative selection and Treg induction (10, 11).

## The Proteome of Human Late-Stage mTECs and HCs Displays a Pro-Inflammatory Signature

Therefore, there is an increasing amount of evidence that at least in mice the post-Aire cells and HCs may contribute to the tonic inflammatory signaling in the thymic medulla, which in turn may play a role in the induction of central tolerance. We aimed to complement these findings with data obtained from human thymi and chose to analyze the proteomic pattern during mTEC maturation, as HCs are known to lose their nuclei and related transcriptional machinery. Accordingly, we microdissected three distinct morphological thymic areas (see **Figure S1**): 1) thymic medulla (labeled as mTECs throughout the proteomics section) 2) the epithelial layer immediately surrounding the HCs and characterized by flattened nuclei (labeled as late mTECs) and 3) the HCs to characterize the changes in the mTEC proteome during three consecutive differentiation stages. The mTEC population is likely to include all different mTECs but the mTEC III (i.e. non-mTEC III) whereas the easily distinguishable late-mTECs and HCs correspond to mTEC III and HCs, respectively. The dissection was performed from three thymi of patients (one year 2 months, one year 7 months, and two years 7 months old) undergoing cardiac surgery. The collected samples were analyzed by nano-LC/MS/MS and the raw data processed by MaxQuant, followed by the differential analysis of the detected proteins and pathway analysis for the changed protein groups (see supplementary materials for detailed description). In addition, we compared this data obtained from thymi to the one obtained from three consecutive differentiation stages of human epidermal keratinocytes collected from healthy grown-up individuals: 1) stratum basale 2) stratum spinosum and 3) stratum granulosum + stratum corneum, in order to detect parallels in mTEC vs keratinocyte late-stage differentiation.

We were able to detect 1095 unique proteins in mTECs, 1026 in late mTECs and 880 in the HCs. Samples from all thymic areas were markedly enriched for keratins comprising between 5-7 different keratins among the top ten most abundant proteins in all samples. On the other hand, the peptides of thymocyte-specific markers CD4 and CD8 were undetectable in all samples whereas CD3 was detected at very low levels (four orders of magnitude lower than the top keratins) in two out of nine samples. Thus, although a minor contribution from hematopoietic cells can’t be excluded, the detected proteome mostly reflects the changes occurring in the thymic stromal compartment.

**Figure 1:**
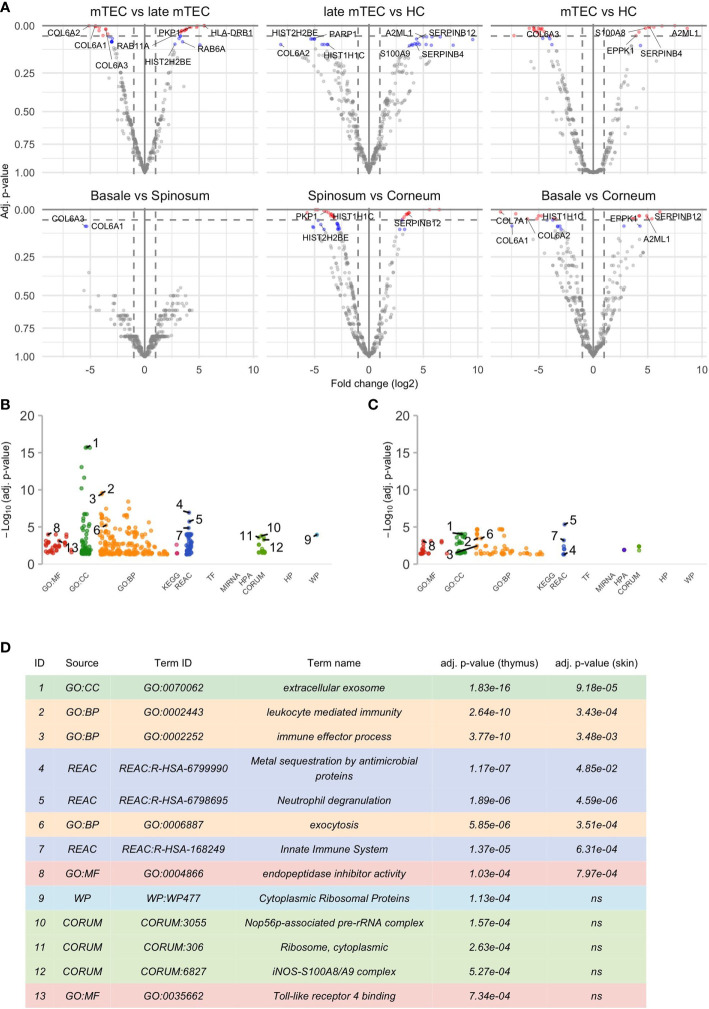
Results of the differential analysis. **(A)** Volcano plots of differential analysis. The x- and y-axis of the volcano plot show log_2_ of fold change (FC) and negative log_10_ of p-values respectively. There are altogether 6 volcano plots for each groupwise comparison shown in the title of the plot. The first source material name in the title always corresponds to the reference group and thus positive log_2_ FC indicates an increase in the later differentiation stage compared to the earlier. Proteins with adj. p-value ≤ 0.05 are colored red, proteins with adj. p-value >0.5 and ≤ 0.1 are colored blue and a selection of them are named by their underlying genes. Only some top genes are shown by names, for full list of genes please see **Table S1**, **S2**. The results of functional enrichment analysis of genes with adj. p-value ≤ 0.1 are visualized by Manhattan plots **(B, C)** that correspond to significant gene sets in the thymus and epidermis respectively. More specifically, these plots convey information about Gene Ontology (GO) with “MF” describing the molecular functions of the gene products, “BP” the biological processes in which they are involved in and “CC” the cellular component where the gene products are located. In addition, there are molecular pathways in which gene sets are enriched in (KEGG, REAC, WP), putative transcription factor binding sites (TF), information about targeted miRNAs (MIRNA), protein complexes (CORUM, HPA) and associated human diseases (HP). For further information please see g:Profiler (https://biit.cs.ut.ee/gprofiler). **(D)** shows further information about the selected GO terms in **(B, C)**. Some highly significant functional terms are not included in **(D)** due to virtual overlap with a functional pathway with even more significant p-value.

Among the unique proteins detected, the keratins, serpins, and S100A family were represented by 42, 14 and 9 members, respectively (**Figure S2**). There was a significant upregulation of 37 and down regulation of 38 proteins during the final stages of mTEC maturation (**Figure 1**; **Table S1**). As expected, the loss of nuclei and compaction of epithelial tissue was reflected by significant downregulation of several proteins with nuclear expression (23 out of 38, **Table S1**) and by downregulation of several proteins known to be highly expressed in the extracellular matrix (**Figures 1** **,** **2**; **Table S1**).

**Figure 2:**
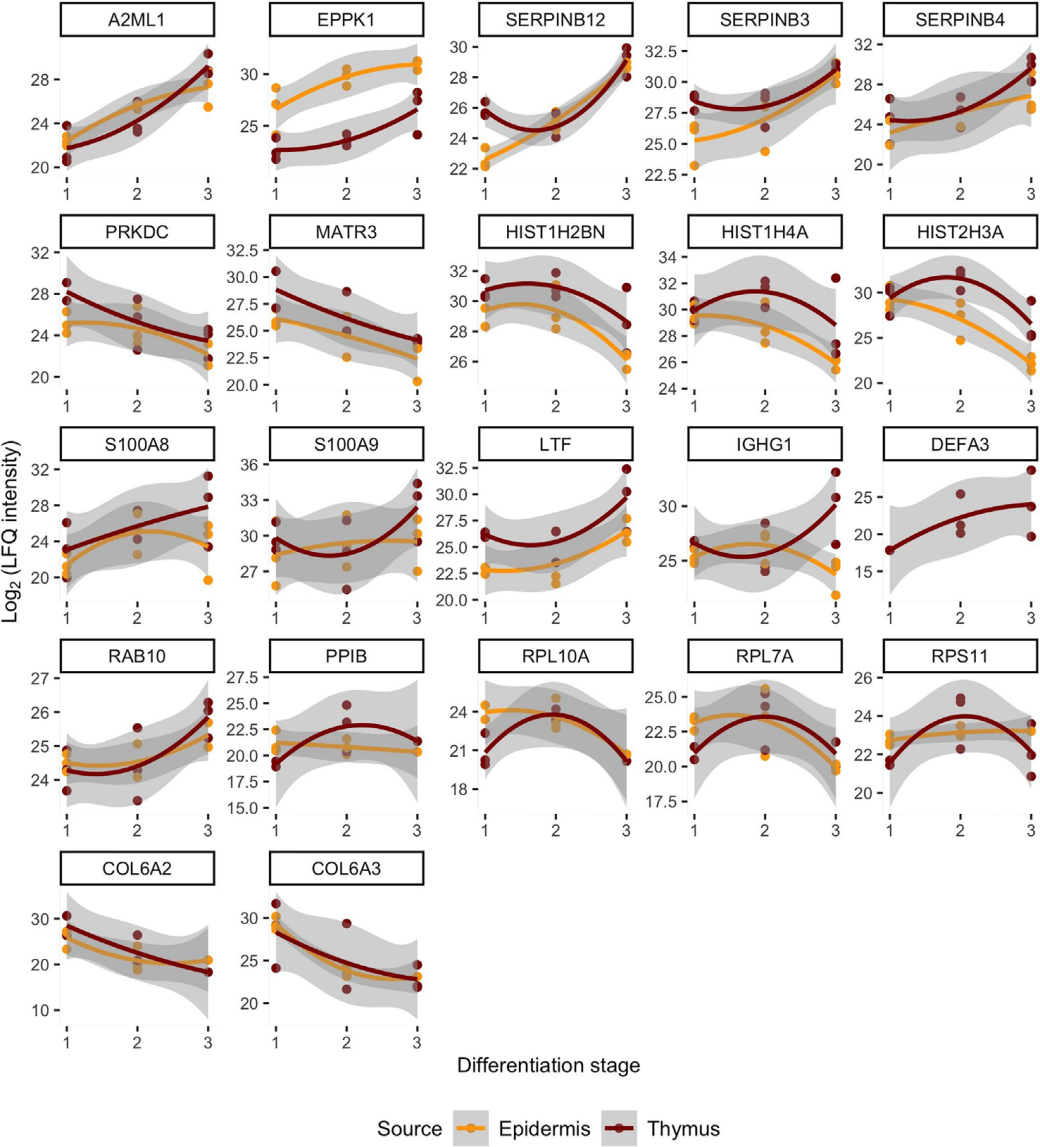
Similarities of protein levels between epidermis and thymus with respect to the differentiation stages and organized based on the localization or function of proteins. The log_2_ intensity levels (LFQ) of 22 proteins are shown in all samples. The x-axis corresponds to the source material that is denoted as differentiation stage. The differentiation stages 1, 2, 3 correspond in case of thymus to the mTEC, late mTEC, HC and in epidermis stratum basale, stratum spinosum and stratum granulosum + stratum corneum. The loess regression lines connect the values in those stages representing the change in protein levels during differentiation. Figure’s first line shows proteins that are more specific to the epidermis, the second line corresponds to the nuclear proteins, the third one to inflammation related proteins, the fourth line to the translation associated proteins and final fifth one to the collagens.

Remarkably, despite the progressive loss of nuclei, the late stages of mTECs were characterized by upregulation of several proteins related to protein translation, folding and intracellular transport (**Figures 1** **,** **2**; **Table S1**). These characteristics also showed up to be significantly altered in the pathway analysis under the cytoplasmic ribosomal complex pathway (**Figure 1**). In addition, there was significant enrichment of proteins involved in exocytosis and extracellular exosome generation (**Figure 1**). As the mTEC-derived exosomes have been shown to carry the keratinocyte-specific autoantigens DSG-1, DSG-3 as well KRT5 and KRT14 (54), the data is compatible with the view suggesting that even after losing their MHCII expression, the post-Aire mTECs and HCs may actively contribute to the induction of central tolerance by synthesis and exocytosis of self-proteins for cross-presentation.

During the final stages of keratinocyte differentiation, we were able to detect 1015 unique proteins in stratum basale, 1092 in stratum spinosum and 718 in stratum granulosum + stratum corneum. Regarding the comparison between the final stages of mTEC and keratinocyte differentiation, we saw several protein groups and pathways being affected similarly in those two different cell types (**Figures 1** **,** **2**; **Table S1**, **S2**). In addition to the expected loss of nuclei and epithelial compaction, the late stages of differentiation were similarly characterized by preferential upregulation of proteins involved in extracellular exosome generation, leukocyte mediated immunity, exocytosis and endopeptidase activity (**Figures 1** **,** **2**; **Table S1**, **S2**). Also, in the thymus and skin, the late stages of differentiation were characterized by increased expression of two auto-antigens, EPPK1 and A2ML1 (**Figures 1** **,** **2**), previously associated with autoimmune skin blistering (55, 56). Altogether, our data confirmed the previously known parallels in mTEC vs keratinocyte differentiation and suggested that at the late stages of differentiation the mTECs can indeed express keratinocyte-specific antigens to be cross-presented by myeloid APCs to the developing thymocytes.

Most importantly, the late-stage mTECs and HCs displayed a clear upregulation of several proteins usually connected to inflammatory processes (**Figures 1** **,** **2**; **Table S1**). This increase in inflammatory proteins was reflected in the pathway analysis showing enrichment for proteins related to leukocyte mediated immunity, immune effector processes and innate immune system (**Figure 1**). Strikingly, among the induced inflammatory proteins were S100A8 and S100A9 and, accordingly, the iNOS-S100A8/A9 complex pathway (**Figure 1**). These S100A proteins, known mainly for their Ca++ binding properties (57), have recently been shown to form a heterodimeric complex, that in turn, through binding and activating TLR4 (57, 58), can induce the expression of several proinflammatory cytokines and mediators. As we also saw an upregulation of the TLR4 binding related processes at the late stages of mTEC differentiation (**Figure 1**), our data strongly suggest that post-Aire mTECs and HCs may play a role in the induction of tonic inflammatory signals by constitutive expression of the endogenous TLR4 agonist, S100A8/A9.

## Discussion

The escape of imperfectly selected T cells from the thymus to the periphery has long been considered as a potential mechanism in the development of autoimmune diseases. The thymocyte selection processes are highly dependent on their cross-talk between different thymic cell populations capable of expressing and presenting ectopic proteins to the developing thymocytes (1, 3). Consequently, the signals behind cellular migration, activation, differentiation and communication in the thymus are of critical importance in the development of T cell repertoire in the periphery.

Therefore, the low-grade constitutive expression of type 1 IFNs in the thymus is highly relevant as both the survival of thymocytes (59) as well as induction of thymic Tregs (60) have been shown to depend on signaling through IFNAR, the receptor for type 1 IFNs. Likewise, the expression of MHC and co-stimulatory molecules on APCs is dependent on inflammatory signals as is their activation and migration to the site of inflammation (61–63). Since TLR4 signaling through IRF3 activation on the other hand is a well-characterized inducer of type 1 IFNs (64), the expression of the endogenous TLR4 agonist, S100A8/A9 by post-Aire mTECs and HCs bears the potential to act as an upstream initiator of a constitutive inflammatory cascade capable of modifying all critical counterparts of the thymic cross-talk.

The proposed role of post-Aire cells/HCs in creating the pro-inflammatory microenvironment by the expression of S100A family members is also supported by previous studies. Thus, in mice the post-Aire cells isolated by using a specific reporter are characterized by high expression of a variety of inflammatory genes, including S100A9 (7), whereas the single cell transcriptomics analysis in humans indicates S100A9 in the top 20 most highly expressed genes in the post-Aire (TEC III) population with several other (DEFB1, ANXA1, CXCL17) inflammation-related genes also belonging to the top 20 list (27).

Alternatively to its role as a TLR4 ligand, S100A8/S100A9 may have intracellular functions in mTECs as they form an LPS-inducible, heterotrimeric complex with iNOS, which elicits S-nitrosylation of GAPDH and a family of other proteins (65) A subsequent relocation of S-nitrosylated GAPDH to the nucleus triggers the cell stress response and apoptosis (66). mTECs constitutively express iNOS, which is upregulated after the contact with self-antigens or with thymocytes activated by TCR stimulation (67), and the expression of S100A8 and S100A9 genes is induced by AIRE (68, 69). We have earlier reported the blockage of AIRE-induced cell death by inhibiting the S-nitrosylation and nuclear translocation of GAPDH (70), suggesting that Aire may mediate the nuclear translocation of GAPDH by so far unknown mechanisms, and induce NO-induced cellular stress and apoptosis in post-Aire mTECs.

Another intriguing topic related to these findings is the previously shown lack of tonic inflammatory signaling in the Aire KO mouse (7) together with the defect in the differentiation of post-Aire populations and the development of autoimmune phenotype (10). Although the role of Aire in regulating mTEC maturation has been established years ago (18, 35), the block in differentiation in Aire KO mouse has mostly been connected to impaired expression of ectopic proteins as a potential mechanism behind the development of autoimmunity. However, as summarized above, there is now an increasing amount of evidence that the developmental block also results in decreased inflammatory signaling in the thymus that seems to be related to the lack of post-Aire mTECs and HCs. As the post-Aire cells and HCs appear immediately after Aire expression during mouse fetal development (34) and are present in high numbers during fetal and perinatal development in humans (42), the local environment modified by these post-Aire populations has the potential to refine the Aire-induced tolerance within the time window when Aire`s effect is the strongest, i.e. during the perinatal stage of development (11, 71). By contrast, another interesting phenomenon, the age-induced inflammatory signaling (26, 32, 72), appears much later when Aire’s expression is severely down-regulated (73) and is, accordingly, less likely to have an effect in Aire-related changes. It remains to be determined whether alteration of the final steps in mTEC differentiation or fine-tuning of the inflammatory thymic microenvironment proves to be a useful target for the treatment of autoimmunity caused by Aire-deficiency or possibly for other defects in central tolerance induction.

## Data Availability Statement

The raw data supporting the conclusions of this article will be made available by the authors, without undue reservation.

## Ethics Statement

The studies involving human participants were reviewed and approved by Ethics Review Committee (ERC) on Human Research of the University of Tartu 170/T-i 28.04.2008. Written informed consent to participate in this study was provided by the participants’ legal guardian/next of kin.

## Author Contributions

ML contributed to study design, analysis, presentation, and wrote the paper. AS contributed to data analysis and presentation. AK, KR, and RB contributed to collection of the samples. HP contributed to data analysis and presentation. PP contributed to study design and presentation. All authors contributed to the article and approved the submitted version.

## Funding

This work was supported by the Estonian Research Council: grant PRG377 and grant PSG59; the European Union through the European Regional Development Fund (Project No 2014‐2020.4.01.15-0012); and the Centre of Excellence in Genomics (EXCEGEN) framework), the University of Tartu Center of Translational Genomics (SP1GVARENG).

## Conflict of Interest

The authors declare that the research was conducted in the absence of any commercial or financial relationships that could be construed as a potential conflict of interest.
