# Supplementary material for: Post-Aire Medullary Thymic Epithelial Cells and Hassall’s Corpuscles as Inducers of Tonic Pro-Inflammatory Microenvironment
Source: Front Immunol. 2021 Apr 2;12:635569. doi: 10.3389/fimmu.2021.635569 (PMC8050345; doi:10.3389/fimmu.2021.635569)
Supplement: Supplementary file 1 [file DataSheet_1.docx]

**Supplementary methods**

**Microdissection**

Fresh samples from the thymic and skin samples were frozen using frozen section medium Neg 50^TM^ (Richard-Allan Scientific) and stored at -80-C^o^ until further processing. After thawing, 10μm were cut by cryostat-microtome and mounted on poly-L-lysin treated glasses. Immediately thereafter, the sections were stained with hematoxylin/eosin and processed by PALM Microbeam laser microdissector (Carl Zeiss Microimaging GmbH). The thymic samples were microdissected from the following morphological areas: 1) middle of the medulla 2) nucleated cells immediately surrounding the HCs 3) HCs. The skin samples were microdissected from: 1) stratum basale 2) stratum spinosum and 3) stratum granulosum + stratum corneum. The dissected areas between comparison groups were kept as close as possible and were (10^6^ μm^2^, mean with sem) 3.6±1.5, 3.6± 1.6, 3.6 ±1.6 for mTECs, late mTECs and HCs, respectively and 1.1±003, 1.1±0.08 and 1.1±0.04 for stratum basale, stratum spinosum and stratum granulosum + stratum corneum, respectively.

**Proteomics**

**Nano-LC/MS/MS measurement**. Proteomics analysis was performed at the Proteomics Core Facility of the University of Tartu. Collected samples were injected to an Ultimate 3000 RSLCnano system (Dionex) using a C18 trap-column (Dionex) and an in-house packed (3 µm C18 particles, Dr Maisch) analytical 50 cm x 75 µm emitter-column (New Objective). Peptides were eluted at 200 nl/min with an 8-40% B 60 min gradient (buffer B: 80% acetonitrile + 0.1% formic acid, buffer A: 0.1% formic acid) to a Q Exactive Plus (Thermo Fisher Scientific) mass spectrometer (MS) using a nano-electrospray source (spray voltage of 2.5 kV). The MS was operated with a top-5 data-dependent acquisition strategy. Briefly, one 350-1400 m/z MS scan at a resolution setting of R=70 000 at 200 m/z was followed by higher-energy collisional dissociation fragmentation (normalized collision energy of 26) of 5 most intense ions (z: +2 to +6) at R=17 500. MS and MS/MS ion target values were 3e6 and 5e4 with 50 and 100 ms injection times, respectively. Dynamic exclusion was limited to 30 s.

**Raw Data Processing**. Mass spectrometric raw files were processed with MaxQuant software package (version 1.5.6.5). Methionine oxidation, glutamine/asparagine deamidation and protein N-terminal acetylation were set as variable modifications, while cysteine carbamidomethylation was defined as a fixed modification. Search was performed against UniProt ([www.uniprot.org](http://www.uniprot.org/)) human reference proteome database using the tryptic digestion rule (including cleavages after proline). Only identifications with minimally 1 peptide 7 amino acids long were accepted and transfer of identifications between runs was enabled. Peptide-spectrum match and protein false discovery rate (FDR) was kept below 1% using a target-decoy approach. All other parameters were default.

**Statistical analysis.** Statistical analysis was performed in R (version 4.0.2). The differential analysis was done with R package “limma” (1) on intensity levels (LFQ) and since the samples were obtained from the same individuals, therefore, the individual to individual variation was also controlled. The obtained p-values were adjusted for multiple testing correction with false discovery rate (FDR) method. Due to the small samples size (3 in each group), all genes with p-value ≤ 0.1 were included for subsequent gene enrichment analysis that was done via g:Profiler R package (2). Latter was also used to visualise enrichment results as Manhattan plots. Euclidean distance was used for clustering and all the heatmaps and upset plots were constructed using R package “ComplexHeatmap”(3). Venn diagrams were produced via R package “VennDiagram”.

Citations:

1. Ritchie, M.E., Phipson, B., Wu, D., Hu, Y., Law, C.W., Shi, W., and Smyth, G.K. (2015). limma powers differential expression analyses for RNA-sequencing and microarray studies. Nucleic Acids Research 43(7), e47.
2. Liis Kolberg and Uku Raudvere (2020). gprofiler2: Interface to the 'g:Profiler' Toolset. R package version 0.2.0. <https://CRAN.R-project.org/package=gprofiler2>
3. Gu, Z. (2016) Complex heatmaps reveal patterns and correlations in multidimensional genomic data. Bioinformatics.
4. Hanbo Chen (2018). VennDiagram: Generate High-Resolution Venn and Euler Plots. R package version 1.6.20. https://CRAN.R-project.org/package=VennDiagram

**Supplementary figure legends**

**Fig S1.** Microdissected areas of the thymic samples. Example of morphological areas from where microdissection was performed: 1 – mTEC, 2 – late mTEC, 3 – Hassall’s corpuscles

**Fig S2. Overview of the data and heatmaps of detected keratins, serpins and S100A family proteins.** (A) a PCA plot showing thymic samples included in the analysis. Each individual sample is coloured according to the source material and is named by the individual from whom the specimen was obtained. This PCA is based on the LFQ intensity levels of 147 proteins and its PC2 separates samples based on source materials. (B) shows smoothed histograms (density plots) of the counts of detected peptides per protein for each source material separately. Those distributions are right-skewed and thus indicating that peptide counts are mostly very low (note the logarithmic x-axis). Clustered heatmaps and upset plots based on peptide counts of detected keratins (A, D), serpins (C, E) and S100A family proteins (E, F). Heatmaps reveal high similarity in counts of detected proteins among all of the samples. Upset plots represent the overlaps of detected proteins (count >= 1 included). There seems to be only marginal differences between samples with late mTEC-s having the highest number of unique keratins and S100 family proteins while Hassall’s corpuscles seem to have the most diverse set of serpins.

**Fig S3. Similarities between epithelial cell differentiation in thymus and epidermis.** (A) heatmap based on proteins intensities (LFQ). Only proteins with adj. p-value ≤ 0.1 from any of the differential analyses were selected. The row annotation tracks show the individual, tissue and the source material where the samples were obtained. There are also 6 column annotation tracks of which each of them corresponds to the separate differential analysis. The grey indicates no significant change (at α = 0.1) while red and blue correspond to up- and downregulation in respect to the reference group respectively. A clustering based on the tissue is relatively evident from this plot. However, Hassall’s corpuscles and stratum corneum seem to cluster still separately. (B-F) Venn diagrams of significantly changed proteins: upregulated (B), downregulated (D) and combined (F). (E-G) are visualization of simulation experiments of the overlap sizes. More specifically, the same number of genes as were significant in the differential analysis (at α = 0.1) were sampled 10 000 times from thymus and epidermis. Then the overlap of those sets was calculated and used as a test statistic. The simulated test statistic values were plotted as histograms and red dashed lines show the actual overlap size. Since in all cases actual overlaps were significantly higher than the simulated ones this again further indicates very similar differentiation dynamics of thymus epithelial cells and epidermis.

**Downregulated in mTEC differentiation (38)** **Upregulated in mTEC differentiation (37)**

| **Inflammation**  **related (14)** | **Protein synthesis/**  **transport/**  **folding (9)** |
| --- | --- |
| HLA-DRB1 | PPIB |
| DEFA3 | RAB11A |
| PRDX1 | RAB11B |
| IGHG2 | RPL7A |
| S100A8 | RPL10A |
| IGKC | RPS9 |
| EPX | RPS11 |
| LCN2 | RPS13 |
| IGHG1 | RAB6A |
| PDIA3 | PDIA3 |
| S100A9 |  |
| IGLL5 |  |
| AZU1 |  |
| C3 |  |
|  |  |
| **Skin**  **development/**  **differentiation (7)** | **Others (7)** |
| SERPINB12 | SERPINA1 |
| SERPINB4 | SLC25A3 |
| PKP1 | SLC25A5 |
| A2ML1 | LMNB2 |
| EPPK1 | GDA |
| SBSN | LCP1 |
| KRT79 | COX6C |

| **Nuclear proteins (23)** |  |
| --- | --- |
| HNRNPF | TARDBP |
| TRIM28 | DDX17 |
| SMC1A | DNAJC8 |
| FBL | DHX9 |
| HIST1H1D | HNRNPA1 |
| CPSF6 | HNRNPA0 |
| NONO | HMGB2 |
| PRKDC | HIST2H2BE |
| HNRNPA3 | HIST2H3A |
| TMPO | PARP1 |
| MATR3 | HIST1H1C |
| HNRNPAB |  |
|  |  |
| **Collagen/**  **connective tissue (5)** | **Others (4)** |
| COL6A2 | PFKL |
| COL6A1 | TCP1 |
| COL6A3 | VCP |
| TNC | TLN1 |
| TGFBI | RPS15 |
|  | FH |
|  | EIF4H |
|  | SLC25A5 |
|  | RPL35 |
|  | HSP90AB1 |

**Table S1.** The list of proteins that were either down- or upregulated during mTEC maturation organized according to their primary expression site or function. Only the proteins whose expression was changed between two consecutive differentiation stages (i.e. between mTEC vs late mTEC or between late mTEC vs HC) are shown.

**Downregulated in ker. differentiation (39) Upregulated in ker. differentiation (17)**

| **Nuclear proteins (15)** | **Collagen/**  **connective tissue/**  **ECM (4)** |
| --- | --- |
| HIST2H3A | COL6A3 |
| HIST1H1C | COL6A1 |
| HIST1H4A | COL7A1 |
| HIST1H2AJ | COL6A2 |
| HIST1H2BN |  |
| HIST2H3PS2 | **Others (15)** |
| HIST2H2BE | EPRS |
| H2AFY | CYB5R1 |
| HNRNPU | AHNAK2 |
| HNRNPC | SPTBN2 |
| ILF2 | SLC25A5 |
| XRCC6 | GPNMB |
| MATR3 | IGHG1 |
| PRKDC | HSPD1 |
| LMNA | ATP5A1 |
|  | PRDX1 |
|  | PRDX6 |
|  | RPL7A |
|  | RPS15A |
|  | RPL22 |
|  | RPS13 |
| **Skin**  **development/**  **differentiation (5)** |  |
| CTNND1 |  |
| PKP1 |  |
| CTNND1 |  |
| CTNNA1 |  |
| GPNMB |  |

| **Skin**  **development/**  **differentiation (11)** | **Others (6)** |
| --- | --- |
| SERPINB12 | AZGP1 |
| SERPINB3 | PGAM1 |
| A2ML1 | ATP6V0C |
| GSDMA | HBA1 |
| DSC1 | CAT |
| CASP14 | LTF |
| TGM3 |  |
| BLMH |  |
| EPPK1 |  |
| HAL |  |
| ASPRV1 |  |

**Table S2.** The list of proteins that were either down- or upregulated during keratinocyte (ker) maturation organized according to their primary expression site or function. Only the proteins whose expression was changed between two consecutive differentiation stages (i.e. between stratum basale vs stratum spinosum or between stratum spinosum vs stratum granulosum + stratum corneum) are shown.
