# Supplementary figures and images for: Post-Aire Medullary Thymic Epithelial Cells and Hassall’s Corpuscles as Inducers of Tonic Pro-Inflammatory Microenvironment
Source: Front Immunol. 2021 Apr 2;12:635569. doi: 10.3389/fimmu.2021.635569 (PMC8050345; doi:10.3389/fimmu.2021.635569)

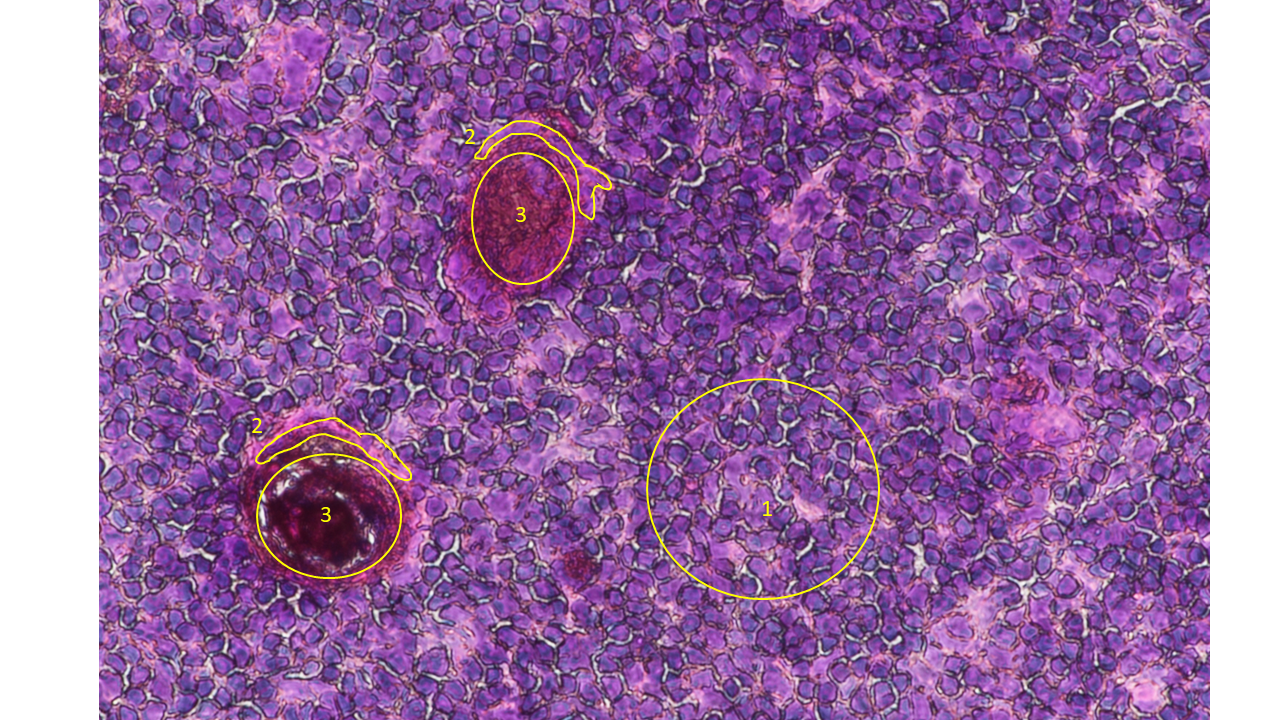

Supplement: Supplementary file 2 [file Image_1.png]

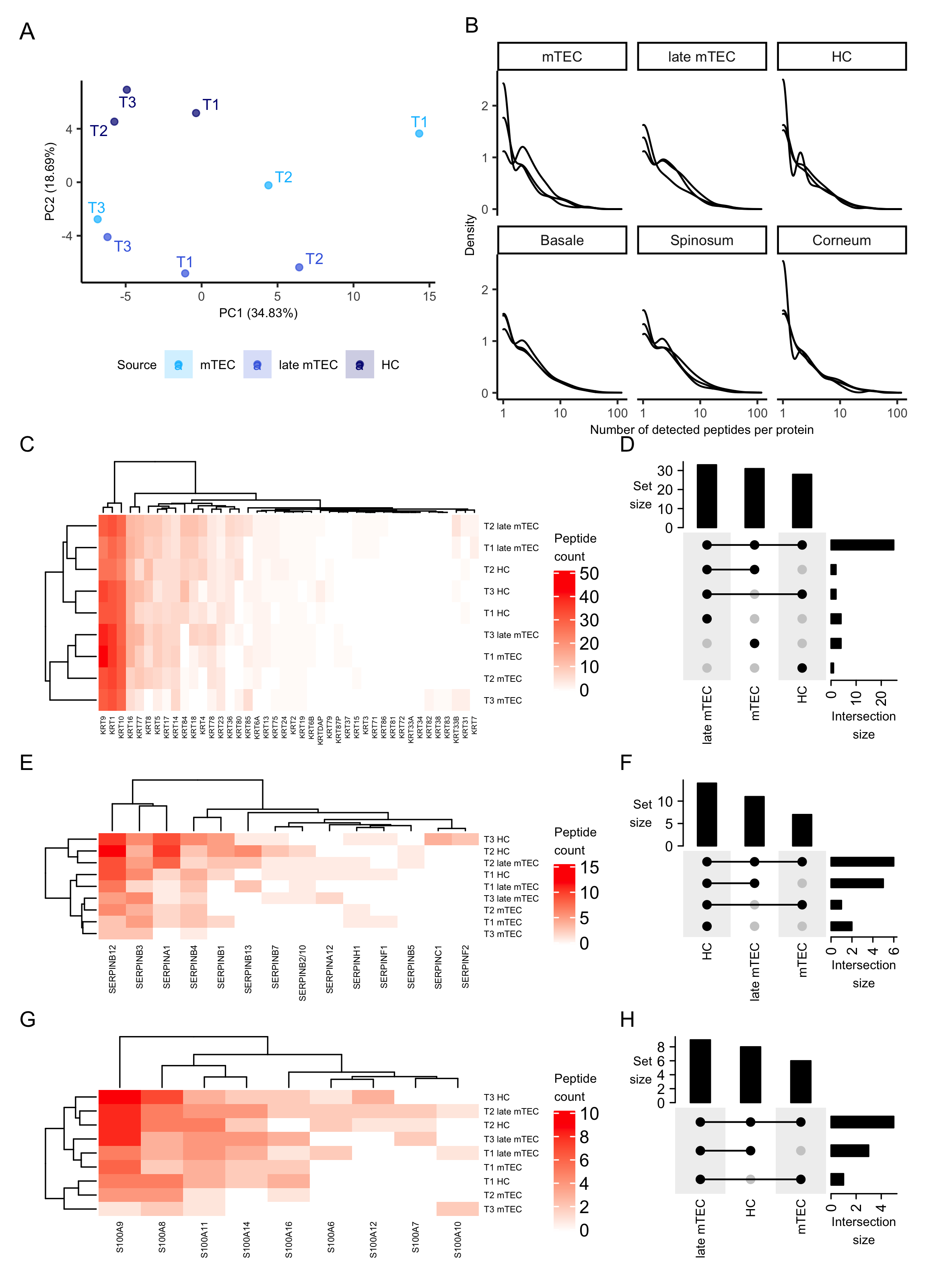

Supplement: Supplementary file 3 [file Image_2.png]

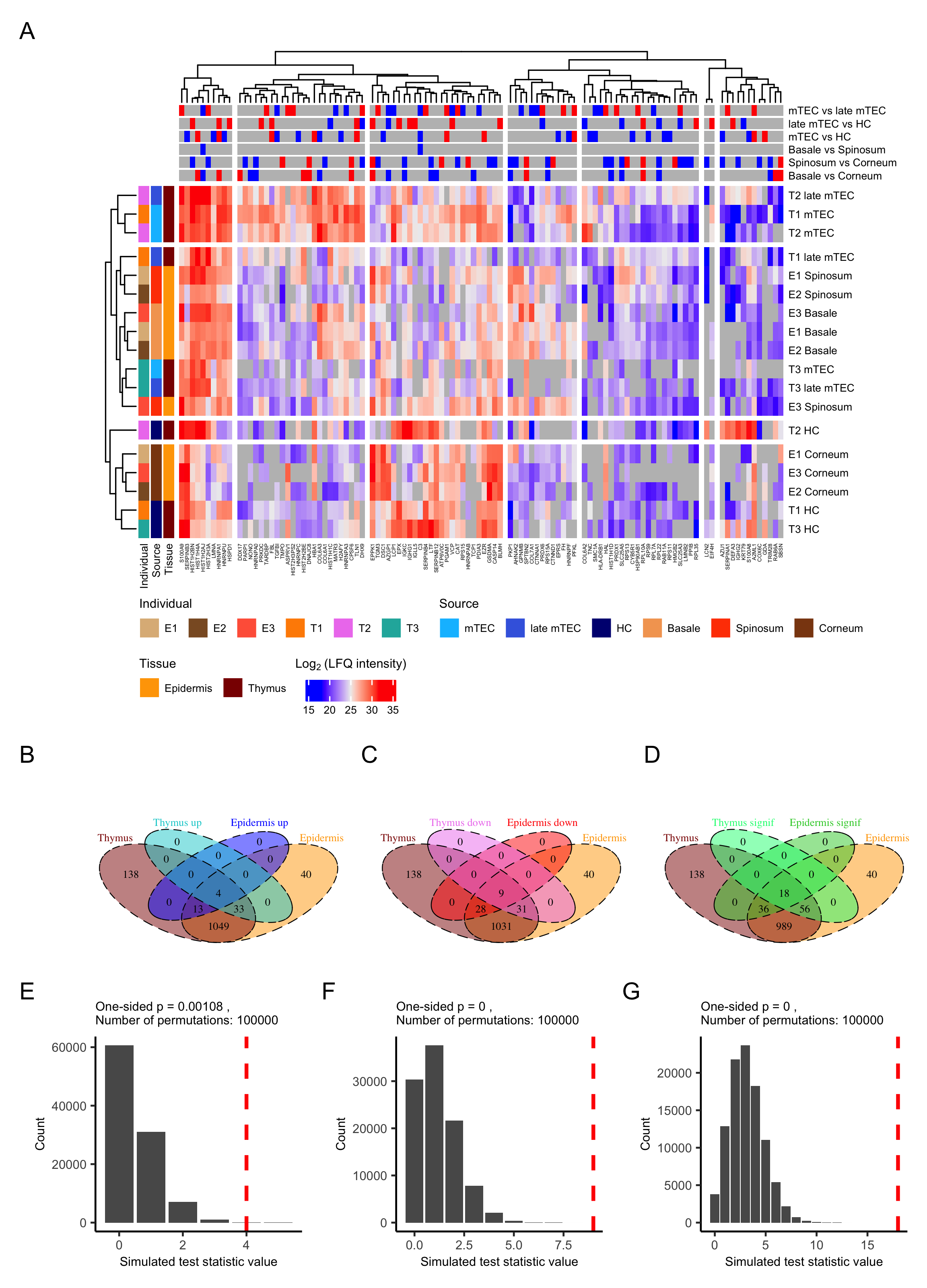

Supplement: Supplementary file 4 [file Image_3.png]
